# Functional connectivity and structural changes of thalamic subregions in episodic migraine

**DOI:** 10.1186/s10194-022-01491-z

**Published:** 2022-09-10

**Authors:** Ying Yang, Huang Xu, Ziru Deng, Wenwen Cheng, Xiuxiu Zhao, Yan Wu, Yuhua Chen, Gui Wei, Ying Liu

**Affiliations:** 1 Department of Radiology, The First Affiliated Hospital of USTC, Division of Life Sciences and Medicine, University of Science and Technology of China, Lujiang Road 17, Hefei, 230001 China; 2 Department of Neurology, The Second Affiliated Hospital of Anhui Medical University, No. 678 FuRong Road, Hefei, 230601 Anhui Province China; 3 Anhui Provincial Stereotactic Neurosurgical Institute, The First Affiliated Hospital of USTC, Division of Life Sciences and Medicine, University of Science and Technology of China, Lujiang Road 17, Hefei, 230001 China; 4 Department of Neurology, The First Affiliated Hospital of USTC, Division of Life Sciences and Medicine, University of Science and Technology of China, Lujiang Road 17, Hefei, 230001 China

**Keywords:** Migraine, Thalamus, Subregions, Functional MRI, High-resolution T1-weighted MRI, DTI

## Abstract

**Background:**

The thalamus plays a crucial role in transmitting nociceptive information to various cortical regions involving migraine-related allodynia and photophobia. Abnormal structural and functional alterations related to the thalamus have been well established. However, it is unknown whether the brain structure and function of the thalamic subregions are differentially affected in this disorder. In this study, we aimed to clarify this issue by comparing the structure and function of 16 thalamic subregions between patients with episodic migraine (EM) and healthy controls (HCs).

**Methods:**

Twenty-seven patients with EM and 30 sex-, age- and education-matched HCs underwent resting-state functional and structural magnetic resonance imaging scans. Functional connectivity (rsFC), grey matter volume (GMV), and diffusion tensor imaging (DTI) parameters of each subregion of the thalamus were calculated and compared between the two groups. Furthermore, correlation analyses between neuroimaging changes and clinical features were performed in this study.

**Results:**

First, compared with HCs, patients with EM exhibited decreased rsFC between the anterior-medial-posterior subregions of the thalamus and brain regions mainly involved in the medial system of the pain processing pathway and default mode network (DMN). Second, for the whole thalamus and each of its subregions, there were no significant differences in GMV between patients with EM and HCs (*P* > 0.05, Bonferroni corrected). Third, there was no significant difference in DTI parameters between the two groups (*P* > 0.05). Finally, decreased rsFC was closely related to scores on the Hamilton Rating Scale for Anxiety (HAMA) and Big Five Inventory (BFI) scales.

**Conclusion:**

Selective functional hypoconnectivity in the thalamic subregions provides neuroimaging evidence supporting the important role of thalamocortical pathway dysfunction in episodic migraine, specifically, that it may modulate emotion and different personality traits in migraine patients.

## Background

Migraine is a highly prevalent neurological disorder that affects over 1 billion people worldwide [1]. A migraine attack comprises moderate-to-severe intensity headache, with a combination of nausea, vomiting, and hypersensitivities to visual, auditory, olfactory, and somatosensory stimuli [2]. However, the neural basis of migraine remains poorly understood. Previous studies have highlighted the importance of the thalamus in migraine pathology. The thalamus is considered the relay centre for transmitting nociceptive information via the trigeminovascular pain pathway from lower brain areas to various cortical regions [3, 4]. Given its widespread connections between the midbrain and cortex, the thalamus is involved in a wide range of functions, including pain modulation, the sleep–wake cycle, awareness, cognitive and emotional behaviours, and the modulation of visual information [5, 6]. Its heterogeneous functionality is based on the fact that the thalamus is a multifactorial construct. Identifying the structural and functional connectivity changes in each thalamic subregion may help clarify the neural underpinnings of migraine.

Resting-state functional magnetic resonance imaging (fMRI) has emerged as a noninvasive imaging technique for measuring spontaneous brain activity based on blood oxygen level-dependent signals in vivo [7, 8]. Resting-state functional connectivity (rsFC), which reflects correlations of activity between brain areas, is a fundamental tool for characterizing brain network alterations [9–11]. With advanced fMRI techniques, the rsFC of the human thalamus has been investigated in health and disease, most extensively in patients with migraine [8, 12]. Based on previous fMRI findings, the thalamus is mostly connected to brain regions involved in pain encoding and visual processing during and between spontaneous attacks [13, 14].

The thalamus is a region with high heterogeneity in cytoarchitecture, connectivity and functionality [3, 6]. Several lines of clinical and experimental evidence have strongly suggested that the posterior, lateral posterior/dorsal, and ventroposteromedial (VPM) thalamic nuclei are likely involved in migraine pathophysiology. Using rsFC analysis, abnormal connectivity between the posterior thalami and various prefrontal cortical areas has been reported in the interictal state, suggesting that pain modulation is disrupted in migraine [15]. The functional connectivity is altered in migraine patients with allodynia between the two bilateral posterior thalami and brain regions involved in emotional-cognitive pain processing and regulation (i.e., limbic, parieto-occipital and temporoparietal brain regions, and the medial prefrontal cortex) [16]. A recent study using fMRI in migraineurs without aura showed reduced FC between the anterior dorsal thalamic nucleus and left precuneus and between the ventral posterior nucleus and left precuneus, right inferior parietal lobule and right middle frontal gyrus [17]. Moreover, in rats, noxious stimulation of the dura was used to reveal ascending projections from brainstem trigeminovascular neurons to higher-order neurons in the posterior and VPM thalamic nuclei [18].

Structural neuroimaging methods have also been used to investigate structural abnormalities of the thalamus in patients with migraine. A multicentre imaging study using high-resolution T1-weighted MRI showed morphological thalamic abnormalities in a large cohort of patients with EM compared with healthy subjects [19]. With the diffusion tensor imaging technique (DTI), previous studies have also reported changes in fractional anisotropy (FA) and mean diffusivity (MD) values in the bilateral thalami in migraine patients without aura versus controls [20, 21].

However, in patients with migraine, the subtler distinctions of the thalamic subregions in communication with the cerebral cortex are still elusive. Therefore, in the current study, we hypothesized that the thalamic subregions are not uniformly impaired in patients with migraine. To address this hypothesis, we conducted a systematic study investigating the functional connectivity and structure of each thalamic subregion in patients with interictal episodic migraine (EM) and healthy controls (HCs) using a combined analysis of multimodal MRI data. Based on connection properties derived from multimodal neuroimaging techniques, the thalamus was subdivided into 16 subregions in vivo. Using high-resolution structural and functional MRI, FC maps, VBM, and DTI parameters of the 16 thalamic subregions were calculated and compared between the two groups. Finally, correlation analyses between neuroimaging changes and clinical features were performed in this study.

## Methods

### Participants

Between November 2021 and April 2022, a total of fifty-seven right-handed individuals were enrolled in the present study, including 27 patients with EM—20 without aura (MWoA) and 7 with aura (MWA)—recruited consecutively from the Headache Clinic of First Affiliated Hospital of University of Science and Technology of China (USTC; Anhui Provincial Hospital), and 30 HCs recruited from the local community via advertisements. EM (with and without aura) was diagnosed according to the International Classification of Headache Disorders-III (ICHD-III) [22]. No migraine preventive medication was used by the participants in the past 3 months. The inclusion criteria for patients and controls included 18–55 years of age, right-handedness, and Han ethnicity. The exclusion criteria were as follows: (I) presence of other neurological diseases; (II) a history of significant physical or psychiatric illnesses; (III) a history of head injury with loss of consciousness; and (IV) pregnancy or any contraindications for MRI. To avoid measuring imaging changes associated with acute migraine symptoms, all patients were scanned during an interictal period, at least 72 h after and 24 h prior to a migraine event. The study procedures were approved by the Ethical Committee of First Affiliated Hospital of USTC and complied with the Declaration of Helsinki. Written informed consent was obtained from all participants before study entry.

### Clinical assessment

Demographic information of the participants (including age, sex, years of education) was recorded. Migraine family history, migraine duration, the Headache Impact Test-6 (HIT-6) [23], and a visual analogue scale (VAS) [24] were used to assess the impact of migraine. The 14-item Hamilton Rating Scale for Anxiety (HAMA) [25] and the Beck Depression Inventory, 2nd edition (BDI-II) [26] were applied to assess the anxiety and depression status of the patients. The Montreal Cognitive Assessment (MoCA) [27] was used to evaluate cognitive function. The Big Five Inventory–60 items (BFI) measures personality traits [28]. Several subjects did not undergo the whole clinical assessment due to personal reasons, e.g., low education level or insufficient time.

### MRI acquisition

All the subjects underwent MRI scans on a GE 3.0 T MR system (DISCOVERY MR750, GE Healthcare, Milwaukee, WI, USA) with a 24-channel head coil at the MRI Center of The First Affiliated Hospital of University of USTC. Earplugs were used to reduce scanner noise, and tight but comfortable foam padding was used to minimize head motion. Before the scanning, all subjects were instructed to keep their eyes closed, relax, move as little as possible, think of nothing in particular, and not fall asleep during the scans. High-resolution, three-dimensional (3D), T1-weighted structural images were acquired using a brain volume (BRAVO) sequence with the following parameters: repetition time (TR) = 8.5 ms; echo time (TE) = 3.2 ms; flip angle (FA) = 12°; field of view (FOV) = 256 mm × 256 mm; matrix = 256 × 256; slice thickness = 1 mm, no gap; 144 axial slices; and acquisition time = 240 s. Resting-state BOLD data were acquired using a gradient-echo single-shot echo planar imaging (GRE-SS-EPI) sequence with the following parameters: TR = 2,000 ms; TE = 30 ms; FA = 90°; FOV = 240 mm × 240 mm; matrix = 64 × 64; slice thickness = 4 mm without gap; 36 interleaved axial slices; 240 volumes; and acquisition time = 480 s. DTI data were acquired using a spin‒echo single-shot echo planar imaging (SE-SS-EPI) sequence with the following parameters: TR = 5, 260 ms; TE = 99 ms; FA = 90°; FOV = 220 mm × 220 mm; matrix = 128 × 128; slice thickness = 5 mm; slice gap = 1 mm; 19 axial slices; 25 diffusion gradient directions (b = 1000 s/mm2) plus five b = 0 reference images; and acquisition time = 142 s. In addition, conventional MRI examination was underwent to exclude the subjects with cerebral infarction, malacia, or occupying lesions.

### Definition of thalamic subregions

**Fig. 1:**
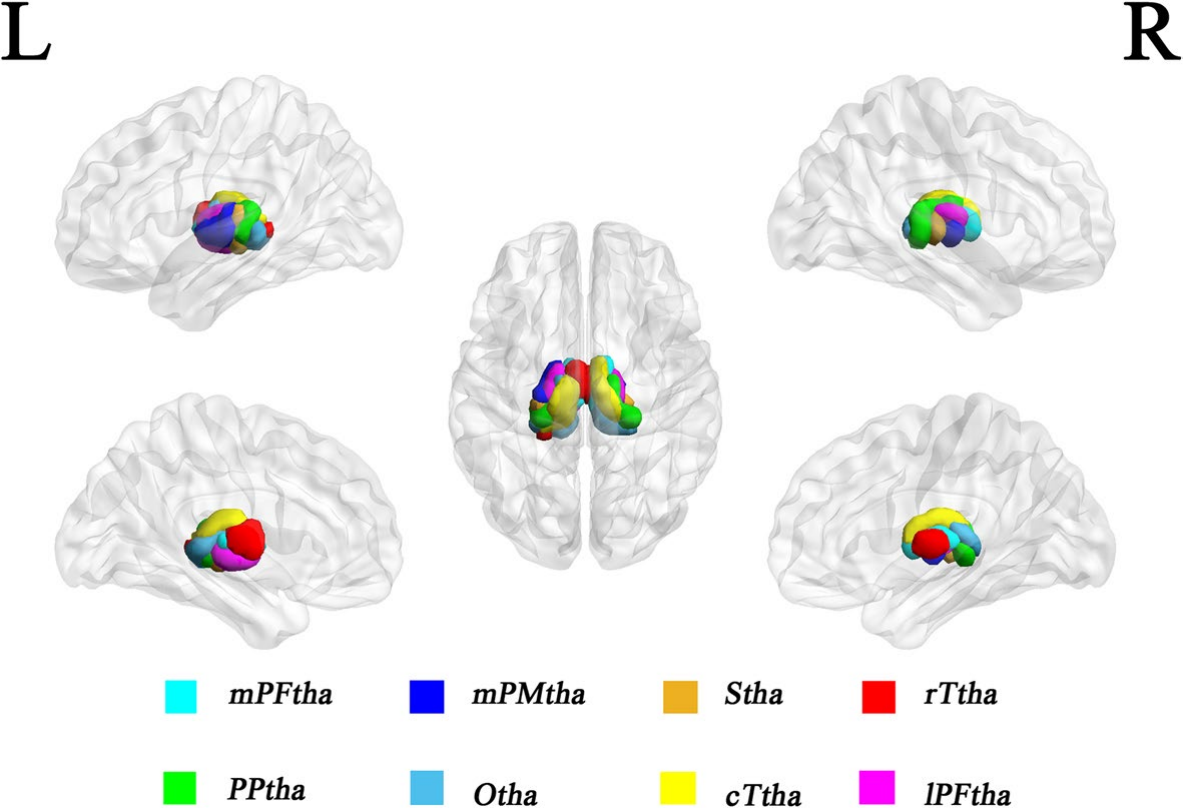
Illustration of subregions of the bilateral thalami. Abbreviations: mPFtha, medial prefrontal thalamus; mPMtha, premotor thalamus; Stha, sensory thalamus; rTtha, rostral temporal thalamus; PPtha, posterior parietal thalamus; Otha, occipital thalamus; cTtha, caudal temporal thalamus; lPFtha, lateral prefrontal thalamus; L, left; R, right

The thalamic subregions were defined according to a connectivity-based parcellation study using multimodal neuroimaging techniques [29]. In each hemisphere, the thalamus was parcellated into the medial prefrontal thalamus (mPFtha), premotor thalamus (mPMtha), sensory thalamus (Stha), rostral temporal thalamus (rTtha), posterior parietal thalamus (PPtha), occipital thalamus (Otha), caudal temporal thalamus (cTtha), and lateral prefrontal thalamus (lPFtha). Thus, we defined a total of 16 regions of interest (ROIs) for the bilateral parts of the thalamus (Fig. 1).

### fMRI data preprocessing

The functional data were preprocessed and analysed using the Statistical Parametric Mapping software (SPM12; http://www.fil.ion.ucl.ac.uk/spm) and the Data Processing and Analysis for Brain Imaging (DPABI_ V3.1_180801; http://rfmri.org/dpabi) [30] in MATLAB R2016b (MathWorks, Inc.) as follows: (1) Removal of the first 10 volumes of the resting-state functional images; (2) Slice timing correction; (3) Head motion correction; (4) Regression of several nuisance covariates (linear drift, estimated motion parameters based on the Friston-24 model, spike volumes with FD > 0.5, white matter signal, and cerebrospinal fluid signal) from the data; (5) Data detrending and bandpass-filtering from 0.01 to 0.1 Hz; (6) Spatial normalization using DARTEL; and (7) Data smoothing with a Gaussian kernel of 6 × 6 × 6 mm^3^ full-width at half-maximum (FWHM). We also calculated FD, which indexes the volume-to-volume changes in head position.

The rsFC analysis was processed using DPABI software (V3.1_180801). For each individual, Pearson's correlation coefficients between the mean time courses of each thalamic subregion and those of each voxel in other parts of the brain were computed. Then, the correlation coefficients were converted into Fisher's z values to improve normality. For each group, individuals' z values were then entered into a random-effect one-sample *t test* in a voxelwise manner to identify brain regions that showed significant positive correlations with each ROI. Finally, a 2-sample *t test* was performed within the positive rsFC mask to quantitatively test group differences in the rsFC of each ROI. Multiple comparisons for these analyses were corrected using a cluster-level familywise error (FWE) method with a corrected threshold of *P* < 0.05.

### GMV calculation

Structural scans were processed using CAT12 (CAT12, http://www.neuro.uni-jena.de/cat/) for SPM12 in MATLAB R2016b for VBM analysis). VBM includes spatial normalization, segmentation and smoothing. In brief, each participant's original T1 image was spatially normalized and segmented into grey and white matter and cerebrospinal fluid (CSF). After data preprocessing, the modulated normalized GMV was smoothed using a 6 mm FWHM Gaussian kernel.

The GMV of each thalamic subregion was extracted and compared between the two groups using the two-sample *t test*. Multiple comparisons were corrected using the Bonferroni method with a significance threshold of *P* < 0.05/16 = 0.003 (16 thalamic subregions). Moreover, the GMV of the whole thalamus was also compared between the two groups, and a *p* value < 0.05 was considered significant.

### DTI analysis

The DTI datasets were preprocessed with the FMRIB Software Library (FSL v6.0.1, https://fsl.fmrib.ox.ac.uk/fsl/fslwiki) [31, 32]. The FSL Diffusion Toolbox (FDT) was used to correct eddy current distortions and head motion. The brain extraction tool (BET) was used to create brain masks from the b0 images. An automated quality control framework was used to assess the diffusion MRI data [33]. AD (axial diffusivity), FA, MD, and RD (radial diffusivity) were calculated by using the FSL toolbox DTIFIT. These images were then coregistered to each subject’s T1-weighted images using the FLIRT linear registration tool, yielding the normalized FA, AD, RD, and MD. Finally, the mean FA, AD, RD, and MD values of each thalamic subregion were extracted and compared between the two groups using the two-sample *t test*. Multiple comparisons were corrected using the Bonferroni method with a significance threshold of *P* < 0.05/16 = 0.003 (16 thalamic subregions).

### Correlations between imaging and clinical parameters

To determine whether thalamic rsFC, GMV, and DTI abnormalities of the thalamic subregions with significant intergroup differences were associated with illness duration and symptom severity (HIT-6, VAS, HAMA, BDI-II, MoCA, and BFI scores), we calculated partial correlations (two‐tailed) in the migraine group after controlling for age, sex, education, TIV, and FD to explore the association between the mean values extracted from each significantly different region and clinical parameters. A *p* value < 0.05 was considered significant.

### Sample size calculation

As our primary goal was to detect differences in the functional connectivity of each thalamic subregion between EMs and HCs, the sample size was calculated based on pilot data from 20 subjects, ten for each group. For the EM group, the rsFC between L-mPMtha and Frontal_Mid_L was 0.13 ± 0.119, between L-rTtha and Frontal_Sup_L was 0.18 ± 0.062, and between L-PPtha and Precuneus_R was 0.25 ± 0.142. For the HC group, the rsFC between L-mPMtha and Frontal_Mid_L was 0.31 ± 0.101, between L-rTtha and Frontal_Sup_L was 0.40 ± 0.135, and between L-PPtha and Precuneus_R was 0.45 ± 0.106. To achieve a desired power of 90% with a significance level of 5%, the required sample size was 10 subjects for each group as calculated by PASS software (https://www.ncss.com/software/pass). For a more conservative estimate, we decided to complete the enrolment when 27 EM and 30 HC subjects had been included in the current study.

## Results

### Demographic, clinical, and MRI characteristics

The main demographic, clinical, and MRI data of all the subjects are listed in Table 1. The patients with EM and HCs did not show significant differences in terms of age, sex, education, or BMI. Moreover, the FD and TIV, including GM, WM, and CSF, of both groups did not show any significant differences. With regard to clinical assessment, migraine patients had significantly higher HAMA scores and lower extraversion domain of BFI scores.

**Table 1:** Demographic, clinical, and MRI characteristics of patients with episodic migraine and HCs

	**Episodic migraine**	**HC**	***P*** **values**
**Demographics**			
N	27	30	
Age	34.89 ± 9.070	35.53 ± 12.153	0.820
Female/male	25/2	30/4	0.467
Education level	13.70 ± 4.697	14.70 ± 4.473	0.416
BMI	22.13 ± 3.091	22.31 ± 2.897	0.818
**MRI characteristics**			
FD (mm)	0.21 ± 0.247	0.13 ± 0.056	0.093
TIV (cm^3^)	1399.8681 ± 111.56465	1426.4777 ± 135.39141	0.425
GM (cm^3^)	606.4202 ± 45.64292	618.1007 ± 50.83728	0.367
WM (cm^3^)	478.5041 ± 44.96136	499.8487 ± 54.89597	0.116
CSF (cm^3^)	314.1511 ± 64.42272	307.7677 ± 58.93003	0.698
**Migraine characteristics**			
Family history	17/27	N/A	
Disease duration (years)	11.11 ± 10.165	N/A	
Aura	7	N/A	
VAS scale	6.37 ± 1.801 (27/27)	N/A	
HIT-6 scale	60.17 ± 6.218 (24/27)	N/A	
HAMA scale	12.43 ± 8.681 (21/27)	4.00 ± 4.520 (29/30)	< 0.001*
BDI-II scale	9.53 ± 11.452 (19/27)	4.38 ± 5.054 (21/30)	0.069
MoCA scale	26.00 ± 3.079 (24/27)	N/A	
BFI scale			
neuroticism	32.71 ± 6.663 (24/27)	30.86 ± 7.901 (21/30)	0.339
extraversion	36.04 ± 5.320 (24/27)	39.90 ± 5.822 (21/30)	0.025*
openness	36.63 ± 7.756 (24/27)	38.29 ± 5.755 (21/30)	0.425
agreeableness	43.04 ± 3.873 (24/27)	44.10 ± 3.285 (21/30)	0.334
conscientiousness	42.46 ± 7.034 (24/27)	44.33 ± 4.004 (21/30)	0.272

*Abbreviations*: *HC* Healthy control, *BMI* Body mass index, *FD* Framewise displacement, *TIV* Total intracranial volume, *GM* Grey matter, *WM* White matter, *CSF* Cerebrospinal fluid, *VAS* Visual Analogue Scale, *HIT-6* Headache Impact Test, *HAMA* Hamilton Rating Scale for Anxiety, *BDI-II* Beck Depression Inventory-II, *MoCA* Montreal Cognitive Assessment, *BFI* Big Five Inventory

Data are shown as the mean ± SD

^*^ *P* < 0.05

### rsFC maps of each thalamic subregion

**Fig. 2:**
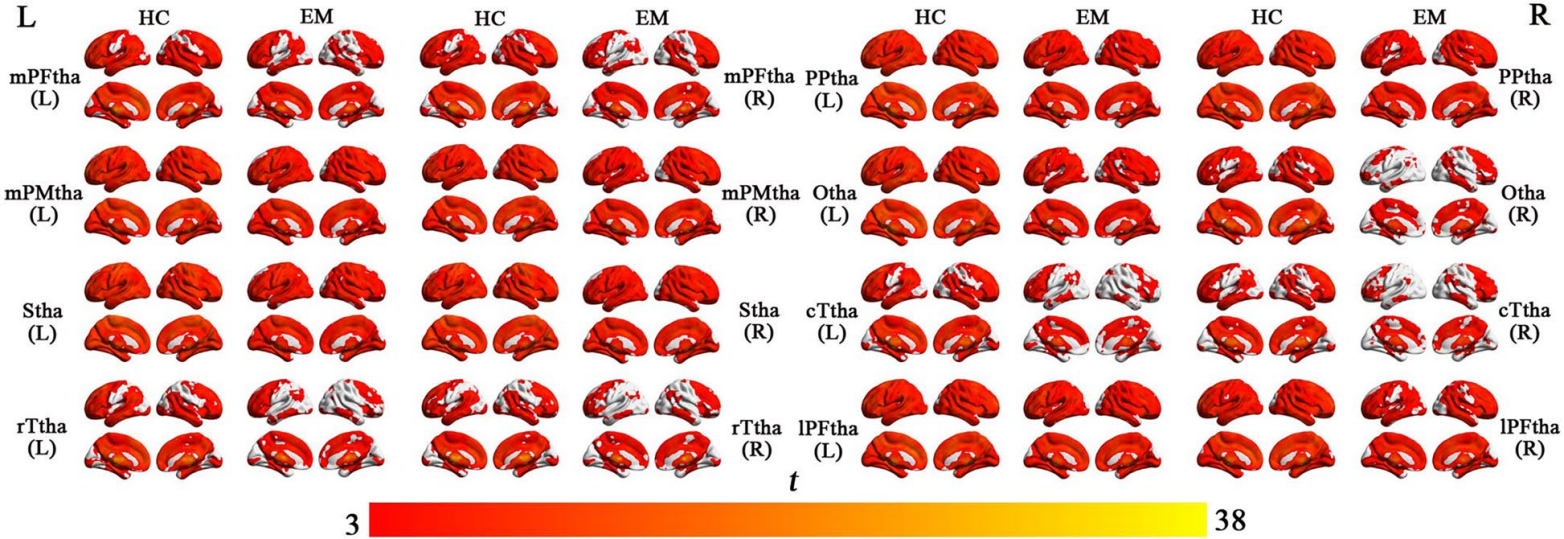
The rsFC maps of the 16 thalamic subregions for both groups. Only the positive rsFC map of each subregion of the thalamus of each group is revealed (*P* < 0.05, cluster-level FWE corrected). Abbreviations: EM, episodic migraine; HC, healthy control; mPFtha, medial prefrontal thalamus; mPMtha, premotor thalamus; Stha, sensory thalamus; rTtha, rostral temporal thalamus; PPtha, posterior parietal thalamus; Otha, occipital thalamus; cTtha, caudal temporal thalamus; lPFtha, lateral prefrontal thalamus; L, left; R, right

The rsFC maps of each thalamic subregion for each group are delineated in Fig. 2 (*P* < 0.05, cluster-level FWE corrected). Overall, visual inspection revealed that patients and controls exhibited similar rsFC patterns in each thalamic subregion, which had significant positive rsFC with widespread brain regions covering almost the entire brain.

### Between-group differences in the rsFC of the thalamic subregions

**Fig. 3:**
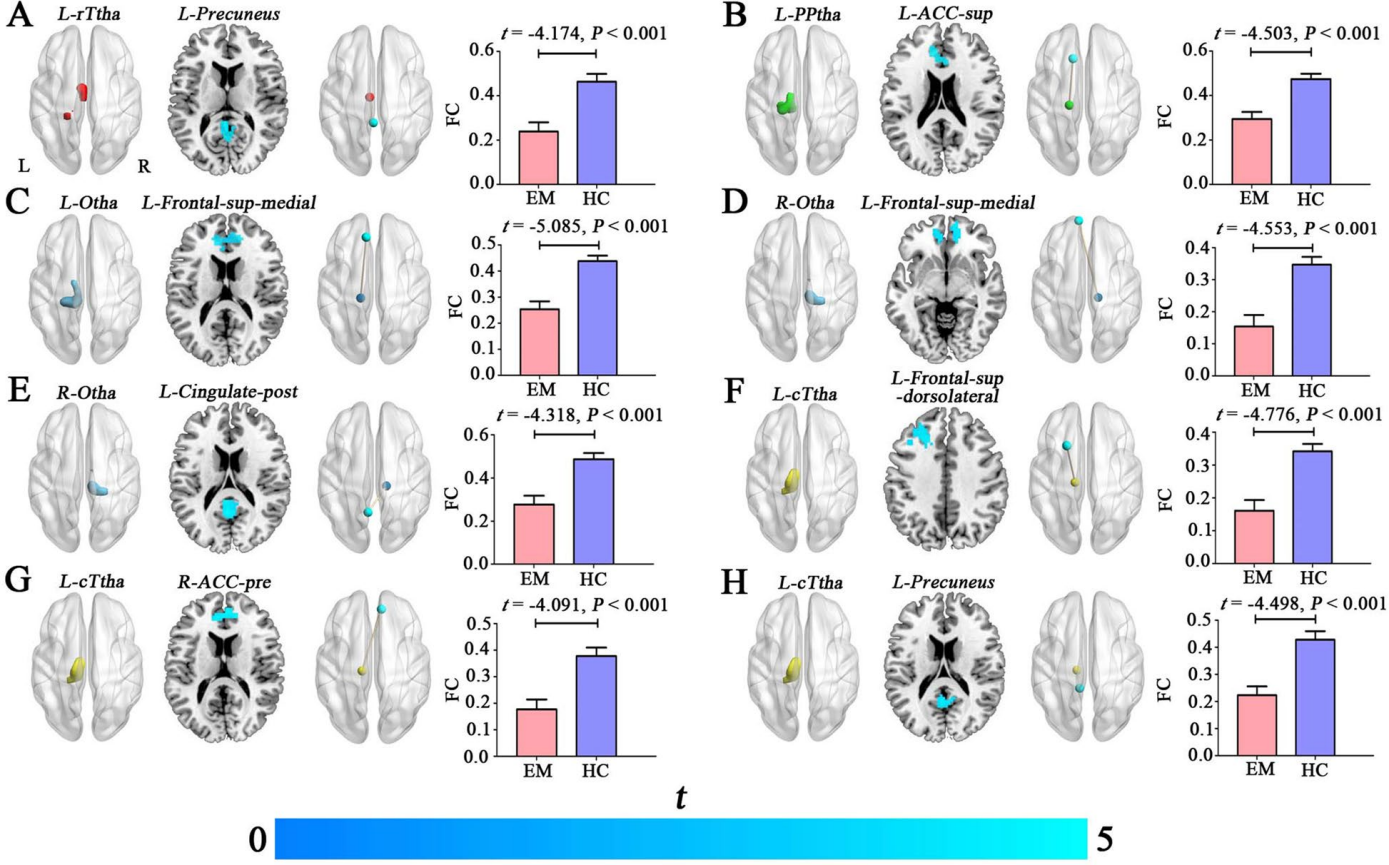
Decreased rsFC of the thalamic subregions in patients with episodic migraine (**A**-**H**). The seed regions (L-rTtha, L-PPtha, L(R)-Otha, L-cTtha) for the voxel-based rsFC are illustrated in the first column. Brain regions (L-ACC-sup, L-Frontal-sup-medial, L-Cingulate-post, L-Frontal-sup, R-ACC-pre, L-Precuneus) showing decreased rsFC with the seed regions in patients with EM compared with HCs are shown in the second column. The functional connectivity (brown lines) between L-rTtha (red sphere) and L-precuneus (light blue sphere), between L-PPtha (light blue sphere) and L-ACC-sup (light blue sphere), between L-Otha (peacock blue sphere) and L-frontal-sup-medial (light blue sphere), between R-Otha (peacock blue sphere) and L-frontal-sup-medial (light blue sphere), between R-Otha (peacock blue sphere) and L-Cingulate-post (light blue sphere), between L-cTtha (yellow sphere) and L-frontal-sup (light blue sphere), between L-cTtha (yelllow sphere) and R-ACC-pre (light blue sphere), and between L-cTtha (yelllow sphere) and L-precuneus (light blue sphere) are shown in the third column. The fourth column represents the ROI-based rsFC analysis for the significant clusters. The error bars indicate the standard error of the mean. Abbreviations: EM, episodic migraine; HC, healthy control; FC, functional connectivity; L, left; R, right

Compared with HCs, patients with EM exhibited significantly decreased rsFC between L-rTtha and L-precuneus, between L-PPtha and L-ACC-sup, between L-Otha and L-frontal-sup-medial, between R-Otha and L-frontal-sup-medial, between R-Otha and L-Cingulate-post, between L-cTtha and L-frontal-sup, between L-cTtha and R-ACC-pre, and between L-cTtha and L-precuneus (*P* < 0.05, cluster-level FWE corrected) (Table 2, Fig. 3). Further ROI-based analyses validated the voxel-based comparison results (Fig. 3).

**Table 2:** Brain regions showing decreased rsFC between the thalamic subregions and cerebral cortex in patients with episodic migraine compared with HCs

Seed regions	Effect regions (AAL-3)	Cluster size (voxels)	Peak *T* values	MNI coordinates (x, y, z)
***L- rTtha***				
Migraine < HC	L-Precuneus	97	4.0993	-3, -42, 9
***L- PPtha***				
Migraine < HC	L-ACC-sup	143	4.2689	-12, 27, 33
***L-Otha***				
Migraine < HC	L-Frontal-sup-medial	404	4.7353	-9, 39, 54
***R-Otha***				
Migraine < HC	L-Frontal-sup-medial	342	4.6943	-9, 60, 12
Migraine < HC	L-Cingulate-post	175	4.4875	0, -45, 24
***L-cTtha***				
Migraine < HC	L-Frontal-sup	245	4.5499	-21, 18, 54
Migraine < HC	R-ACC-pre	112	4.128	9, 45, 18
Migraine < HC	L-Precuneus	243	4.3971	-9, -42, 6

*Abbreviations*: *rTtha* Rostral temporal thalamus, *PPtha* Posterior parietal thalamus, *Otha* Occipital thalamus, *cTtha* Caudal temporal thalamus, *ACC-sup* Anterior cingulate cortex, supracallosal, *Frontal-sup-medial* superior frontal gyrus, medial, *Cingulate-post* Posterior cingulate gyrus, *Frontal-sup* Superior frontal gyrus, dorsolateral, *ACC-pre* Anterior cingulate cortex, pregenual, *MNI* Montreal Neurological Institute, *AAL-3* Automated anatomical labelling atlas-3, *HC* Healthy control, *rsFC* Resting-state functional connectivity, *L* left, *R* Right

### Between-group differences in the GMV of the thalamic subregions

**Fig. 4:**
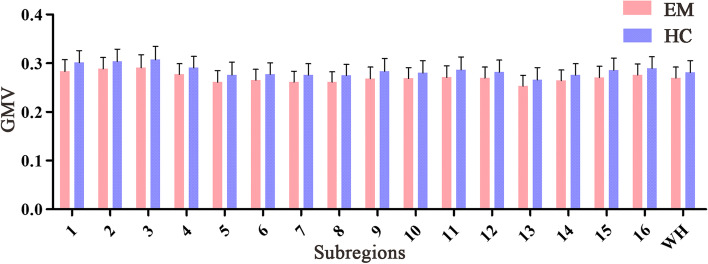
Between-group comparisons of the GMV of the thalamic subregions and whole thalamus. Abbreviations: GMV, grey matter volume (mm^3^); WH, whole thalamus; EM, episodic migraine; HC, healthy control. The error bars indicate the standard deviation. 1–16 represent L(R) mPFtha, L(R) mPMtha, L(R) Stha, L(R) rTtha, L(R) PPtha, L(R) Otha, L(R) cTtha, and L(R) lPFtha, respectively

For all thalamic subregions, there were no significant differences in the GMV between patients with EM and HCs (*P* > 0.05, Bonferroni corrected) (Table 3, Fig. 4). Moreover, we did not observe significant differences in the GMV of the whole thalamus between the two groups (*P* > 0.05).

**Table 3:** GMV changes in the thalamic subregions in patients with episodic migraine compared with HCs

GMV (mm^3^)	Episodic migraine	HC	*T* values	*P* values
*L-mPFtha*	0.28 ± 0.024	0.30 ± 0.025	-2.701	0.009
*R-mPFtha*	0.29 ± 0.023	0.30 ± 0.025	-2.441	0.018
*L-mPMtha*	0.29 ± 0.026	0.31 ± 0.027	-2.242	0.029
*R-mPMtha*	0.28 ± 0.022	0.29 ± 0.023	-2.247	0.029
*L-Stha*	0.26 ± 0.024	0.28 ± 0.026	-2.207	0.031
*R-Stha*	0.27 ± 0.022	0.28 ± 0.024	-1.928	0.059
*L-rTtha*	0.26 ± 0.022	0.28 ± 0.023	-2.298	0.025
*R-rTtha*	0.26 ± 0.021	0.28 ± 0.022	-2.391	0.020
*L-PPtha*	0.27 ± 0.024	0.28 ± 0.026	-2.267	0.027
*R-PPtha*	0.27 ± 0.022	0.28 ± 0.025	-1.811	0.076
*L-Otha*	0.27 ± 0.024	0.29 ± 0.026	-2.307	0.025
*R-Otha*	0.27 ± 0.022	0.28 ± 0.025	-1.867	0.067
*L-cTtha*	0.25 ± 0.022	0.27 ± 0.025	-2.072	0.043
*R-cTtha*	0.26 ± 0.022	028 ± 0.023	-1.901	0.063
*L-lPFtha*	0.27 ± 0.023	0.29 ± 0.025	-2.371	0.021
*R-lPFtha*	0.28 ± 0.022	0.29 ± 0.024	-2.207	0.032
*WH*	0.27 ± 0.022	0.28 ± 0.024	-1.811	0.076

*Abbreviations*: *mPFtha* Medial prefrontal thalamus, *mPMtha* Premotor thalamus, *Stha* Sensory thalamus, *rTtha* Rostral temporal thalamus, *PPtha* Posterior parietal thalamus, *Otha* Occipital thalamus, *cTtha* Caudal temporal thalamus, *lPFtha* Lateral prefrontal thalamus, *WH* Whole thalamus, *HC* Healthy control, *GMV* Grey matter volume, *L* Left, *R* Right

### Between-group differences in DTI parameters (FA, AD, MD, and RD) of the thalamic subregions

There were no significant differences between the two groups in the DTI parameters (FA, AD, MD, and RD) of all 16 thalamic subregions (*P* > 0.05, Bonferroni corrected) (Table 4).

**Table 4:** DTI parameter (FA, AD, RD, MD) changes in the thalamic subregions in patients with episodic migraine compared with HCs

DTI parameters	Episodic migraine	HC	*T* values	*P* values
FA				
*L-mPFtha*	0.35 ± 0.022	0.36 ± 0.022	-0.664	0.509
*R-mPFtha*	0.36 ± 0.023	0.37 ± 0.024	-0.690	0.493
*L-mPMtha*	0.34 ± 0.019	0.34 ± 0.018	-0.411	0.683
*R-mPMtha*	0.35 ± 0.022	0.36 ± 0.024	-0.476	0.636
*L-Stha*	0.35 ± 0.022	0.36 ± 0.022	-0.210	0.835
*R-Stha*	0.38 ± 0.024	0.38 ± 0.025	-0.234	0.816
*L-rTtha*	0.35 ± 0.022	0.35 ± 0.022	-0.508	0.613
*R-rTtha*	0.36 ± 0.023	0.37 ± 0.024	-0.689	0.494
*L-PPtha*	0.35 ± 0.022	0.35 ± 0.021	-0.301	0.764
*R-PPtha*	0.37 ± 0.023	0.37 ± 0.024	-0.186	0.853
*L-Otha*	0.35 ± 0.023	0.35 ± 0.022	-0.413	0.681
*R-Otha*	0.37 ± 0.025	0.37 ± 0.025	-0.331	0.742
*L-cTtha*	0.36 ± 0.023	0.37 ± 0.022	-0.539	0.592
*R-cTtha*	0.37 ± 0.023	0.37 ± 0.023	-0.584	0.562
*L-lPFtha*	0.34 ± 0.021	0.35 ± 0.021	-0.476	0.636
*R-lPFtha*	0.36 ± 0.023	0.37 ± 0.024	-0.401	0.690
AD				
*L-mPFtha*	1.706E-03 ± 1.018E-04	1.719E-03 ± 1.138E-04	-0.469	0.641
*R-mPFtha*	1.649E-03 ± 9.868E-05	1.676E-03 ± 1.051E-04	-1.007	0.318
*L-mPMtha*	1.526E-03 ± 8.538E-05	1.539E-03 ± 9.224E-05	-0.567	0.573
*R-mPMtha*	1.593E-03 ± 8.119E-05	1.628E-03 ± 9.267E-05	-1.477	0.145
*L-Stha*	1.545E-03 ± 8.408E-05	1.562E-03 ± 1.007E-04	-0.699	0.488
*R-Stha*	1.504E-03 ± 7.716E-05	1.536E-03 ± 8.790E-05	-1.477	0.145
*L-rTtha*	1.671E-03 ± 9.859E-05	1.685E-03 ± 1.075E-04	-0.526	0.601
*R-rTtha*	1.725E-03 ± 9.693E-05	1.754E-03 ± 1.084E-04	-1.056	0.296
*L-PPtha*	1.572E-03 ± 9.249E-05	1.580E-03 ± 1.021E-04	-0.316	0.753
*R-PPtha*	1.545E-03 ± 9.033E-05	1.573E-03 ± 9.459E-05	-1.140	0.259
*L-Otha*	1.612E-03 ± 9.745E-05	1.618E-03 ± 1.116E-04	-0.215	0.831
*R-Otha*	1.628E-03 ± 1.076E-04	1.656E-03 ± 1.078E-04	-0.989	0.327
*L-cTtha*	1.644E-03 ± 1.137E-04	1.652E-03 ± 1.114E-04	-0.273	0.786
*R-cTtha*	1.634E-03 ± 1.142E-04	1.661E-03 ± 1.077E-04	-0.905	0.369
*L-lPFtha*	1.643E-03 ± 8.795E-05	1.659E-03 ± 1.020E-04	-0.631	0.531
*R-lPFtha*	1.600E-03 ± 8.662E-05	1.632E-03 ± 9.591E-05	-1.317	0.193
RD				
*L-mPFtha*	1.062E-03 ± 1.051E-04	1.064E-03 ± 1.071E-04	-0.079	0.937
*R-mPFtha*	1.007E-03 ± 1.027E-04	1.017E-03 ± 1.018E-04	-0.380	0.705
*L-mPMtha*	9.557E-04 ± 8.645E-05	9.611E-04 ± 8.249E-05	-0.244	0.808
*R-mPMtha*	9.918E-04 ± 8.383E-05	1.009E-03 ± 8.824E-05	-0.766	0.447
*L-Stha*	9.433E-04 ± 8.687E-05	9.522E-04 ± 9.194E-05	-0.375	0.709
*R-Stha*	8.906E-04 ± 7.857E-05	9.075E-04 ± 8.422E-05	-0.780	0.438
*L-rTtha*	1.033E-03 ± 1.005E-04	1.036E-03 ± 1.004E-04	-0.141	0.889
*R-rTtha*	1.060E-03 ± 1.015E-04	1.072E-03 ± 1.027E-04	-0.452	0.653
*L-PPtha*	9.653E-04 ± 9.411E-05	9.659E-04 ± 9.433E-05	-0.023	0.982
*R-PPtha*	9.215E-04 ± 9.083E-05	9.347E-04 ± 9.190E-05	-0.548	0.586
*L-Otha*	9.953E-04 ± 9.867E-05	9.923E-04 ± 1.020E-04	0.114	0.910
*R-Otha*	9.842E-04 ± 1.084E-04	9.968E-04 ± 1.043E-04	-0.444	0.659
*L-cTtha*	9.935E-04 ± 1.151E-04	9.913E-04 ± 1.057E-04	0.076	0.940
*R-cTtha*	9.789E-04 ± 1.138E-04	9.887E-04 ± 1.039E-04	-0.340	0.735
*L-lPFtha*	1.034E-03 ± 9.193E-05	1.041E-03 ± 9.462E-05	-0.253	0.801
*R-lPFtha*	9.778E-04 ± 9.150E-05	9.936E-04 ± 9.503E-05	-0.638	0.526
MD				
*L-mPFtha*	1.277E-03 ± 1.031E-04	1.283E-03 ± 1.083E-04	-0.213	0.832
*R-mPFtha*	1.221E-03 ± 1.005E-04	1.237E-03 ± 1.017E-04	-0.595	0.554
*L-mPMtha*	1.146E-03 ± 8.542E-05	1.154E-03 ± 8.499E-05	-0.359	0.721
*R-mPMtha*	1.192E-03 ± 8.171E-05	1.215E-03 ± 8.824E-05	-1.021	0.312
*L-Stha*	1.144E-03 ± 8.493E-05	1.155E-03 ± 9.365E-05	-0.492	0.625
*R-Stha*	1.095E-03 ± 7.672E-05	1.117E-03 ± 8.370E-05	-1.035	0.305
*L-rTtha*	1.245E-03 ± 9.904E-05	1.253E-03 ± 1.018E-04	-0.274	0.785
*R-rTtha*	1.281E-03 ± 9.908E-05	1.299E-03 ± 1.034E-04	-0.662	0.511
*L-PPtha*	1.168E-03 ± 9.273E-05	1.171E-03 ± 9.590E-05	-0.124	0.902
*R-PPtha*	1.129E-03 ± 8.961E-05	1.147E-03 ± 9.135E-05	-0.757	0.452
*L-Otha*	1.201E-03 ± 9.733E-05	1.201E-03 ± 1.041E-04	0.001	0.999
*R-Otha*	1.199E-03 ± 1.072E-04	1.217E-03 ± 1.041E-04	-0.634	0.529
*L-cTtha*	1.210E-03 ± 1.140E-04	1.212E-03 ± 1.067E-04	-0.042	0.967
*R-cTtha*	1.197E-03 ± 1.132E-04	1.213E-03 ± 1.041E-04	-0.536	0.594
*L-lPFtha*	1.237E-03 ± 8.974E-05	1.247E-03 ± 9.610E-05	-0.385	0.702
*R-lPFtha*	1.185E-03 ± 8.888E-05	1.207E-03 ± 9.400E-05	-0.872	0.387

*Abbreviations*: *mPFtha* Medial prefrontal thalamus, *mPMtha* Premotor thalamus, *Stha* Sensory thalamus, *rTtha* Rostral temporal thalamus, *PPtha* Posterior parietal thalamus, *Otha* Occipital thalamus, *cTtha* Caudal temporal thalamus, *lPFtha* Lateral prefrontal thalamus, *HC* Healthy control, *DTI* Diffusion Tensor Imaging, *FA* Fractional anisotropy, *MD* Mean diffusivity, *AD* Axial diffusivity, *RD* Radial diffusivity, *L* Left, *R* Right

### Partial correlation analyses between MRI data and clinical scales

After controlling for age, sex, education, and FD, decreased rsFC between L-rTtha and L-Precuneus, between L-PPtha and L-ACC-sup, between R-Otha and L-Cingulate-post, between L-cTtha and L-Frontal-sup, between L-cTtha and R-ACC-pre, and between L-cTtha and L-Precuneus was significantly correlated with greater HAMA in patients with EM (Table 5).

**Table 5:** Partial correlations between decreased rsFC and clinical data in patients with episodic migraine after controlling for age, sex, education, and FD

	Disease duration (years)	VAS	HIT-6	HAMA	BDI-II	MoCA
Decreased rsFC between ***L-rTtha*** and ***L-Precuneus***						
***P(r)***	-0.235	-0.182	-0.145	-0.301	0.173	0.131
***P* value**	0.293	0.418	0.554	0.045*	0.319	0.593
Decreased rsFC between ***L-PPtha*** and ***L-ACC-sup***						
***P(r)***	-0.009	0.074	0.147	-0.389	-0.169	0.125
***P* value**	0.967	0.742	0.547	0.008*	0.333	0.609
Decreased rsFC between ***L-Otha*** and ***L-Frontal-sup-medial***						
***P(r)***	-0.156	-0.065	0.110	-0.286	0.056	0.144
***P* value**	0.489	0.774	0.655	0.057	0.748	0.556
Decreased rsFC between ***R-Otha*** and ***L-Frontal-sup-medial***						
***P(r)***	-0.006	-0.235	-0.177	-0.283	-0.040	0.378
***P* value**	0.980	0.293	0.468	0.060	0.818	0.111
Decreased rsFC between ***R-Otha*** and ***L-Cingulate-post***						
***P(r)***	-0.042	-0.383	-0.358	-0.338	0.060	0.088
***P* value**	0.851	0.079	0.132	0.023*	0.733	0.721
Decreased rsFC between ***L-cTtha*** and ***L-Frontal-sup***						
***P(r)***	0.073	-0.078	0.301	-0.354	0.022	0.372
***P* value**	0.745	0.730	0.210	0.017*	0.898	0.117
Decreased rsFC between ***L-cTtha*** and ***R-ACC-pre***						
***P(r)***	-0.075	0.003	0.012	-0.303	-0.095	0.276
***P* value**	0.741	0.990	0.961	0.043*	0.586	0.253
Decreased rsFC between ***L-cTtha*** and ***L-Precuneus***						
***P(r)***	-0.040	-0.248	-0.069	-0.357	0.047	0.131
***P* value**	0.860	0.266	0.780	0.016*	0.787	0.594

*Abbreviations*: *rTtha* Rostral temporal thalamus, *PPtha* Posterior parietal thalamus, *Otha* Occipital thalamus, *cTtha* Caudal temporal thalamus, *ACC-sup* Anterior cingulate cortex, supracallosal, *Frontal-sup-medial* Superior frontal gyrus, medial, *Cingulate-post* Posterior cingulate gyrus, *Frontal-sup* Superior frontal gyrus, dorsolateral, *ACC-pre* Anterior cingulate cortex, pregenual, *FD* framewise displacement, *VAS* Visual Analogue Scale, *HIT-6* Headache Impact Test, *HAMA* Hamilton Rating Scale for Anxiety, *BDI-II* Beck Depression Inventory-II, *MoCA* Montreal Cognitive Assessment, *rsFC* Resting-state functional connectivity, *L* Left, *R* right

^*^P < 0.05

For BFI scales, lower extraversion was significantly correlated with decreased rsFC between L-Otha and L-Frontal-sup-medial, between R-Otha and L-Frontal-sup-medial, between L-cTtha and L-Frontal-sup, between L-cTtha and R-ACC-pre, and between L-cTtha and L-Precuneus, and lower conscientiousness was significantly correlated with decreased rsFC between L-Otha and L-Frontal-sup-medial in patients with EM. However, we did not observe these significant correlations in the HC group (Table 6).

**Table 6:** Partial correlations between decreased rsFC and BFI in patients with episodic migraine and HCs after controlling for age, sex, education, and FD

	BFI				
	neuroticism	extraversion	openness	agreeableness	conscientiousness
Decreased rsFC between ***L-rTtha*** and ***L-Precuneus (EM/HC)***					
***P(r)***	-0.002/-0.214	0.300/0.206	0.029/0.247	-0.121/-0.327	0.180/-0.271
***P* value**	0.989/0.410	0.060/0.428	0.857/0.340	0.456/0.201	0.267/0.293
Decreased rsFC between ***L-PPtha*** and ***L-ACC-sup (EM/HC)***					
***P(r)***	-0.353/-0.694	0.600/0.645	0.227/0.241	-0.007/0.077	0.327/0.597
***P* value**	0.026*/0.002*	0.000*/0.005*	0.158/0.351	0.966/0.770	0.040*/0.011*
Decreased rsFC between ***L-Otha*** and ***L-Frontal-sup-medial (EM/HC)***					
***P(r)***	-0.203/-0.443	0.477/0.390	0.167/0.220	-0.082/-0.037	0.368/0.422
***P* value**	0.209/0.075	0.002*/0.122	0.304/0.395	0.614/0.887	0.020*/0.091
Decreased rsFC between ***R-Otha*** and ***L-Frontal-sup-medial (EM/HC)***					
***P(r)***	-0.230/-0.238	0.373/0.063	0.055/0.028	0.121/0.068	0.294/0.207
***P* value**	0.154/0.358	0.018*/0.811	0.736/0.916	0.458/0.796	0.066/0.425
Decreased rsFC between ***R-Otha*** and ***L-Cingulate-post (EM/HC)***					
***P(r)***	-0.033/-0.198	0.297/0.024	0.059/0.592	-0.072/-0.184	0.162/-0.126
***P* value**	0.838/0.445	0.063/0.928	0.718/0.012*	0.660/0.479	0.319/0.630
Decreased rsFC between ***L-cTtha*** and ***L-Frontal-sup (EM/HC)***					
***P(r)***	-0.226/-0.498	0.446/0.250	0.145/0.063	0.007/-0.120	0.245/0.111
***P* value**	0.162/0.042*	0.004*/0.332	0.372/0.809	0.964/0.646	0.127/0.670
Decreased rsFC between ***L-cTtha*** and ***R-ACC-pre (EM/HC)***					
***P(r)***	-0.195/-0.187	0.416/0.159	0.121/-0.110	0.013/-0.126	0.199/-0.116
***P* value**	0.229/0.471	0.008*/0.542	0.457/0.675	0.937/0.629	0.218/0.658
Decreased rsFC between ***L-cTtha*** and ***L-Precuneus (EM/HC)***					
***P(r)***	-0.097/-0.285	0.369/0.136	0.138/0.236	-0.108/-0.356	0.180/-0.282
***P* value**	0.553/0.268	0.019*/0.602	0.395/0.363	0.506/0.161	0.267/0.273

*Abbreviations*: *rTtha* Rostral temporal thalamus, *PPtha* Posterior parietal thalamus, *Otha* Occipital thalamus, *cTtha* Caudal temporal thalamus, *ACC-sup* Anterior cingulate cortex, supracallosal, *Frontal-sup-medial* Superior frontal gyrus, medial, *Cingulate-post* Posterior cingulate gyrus, *Frontal-sup* Superior frontal gyrus, dorsolateral, *ACC-pre* Anterior cingulate cortex, pregenual, *FD* Framewise displacement, *BFI* Big Five Inventory, *rsFC* Resting-state functional connectivity, *L* left, *R* Right

^*^P < 0.05

## Discussion

In this study, we systematically investigated the morphology and functional connectivity (FC) changes in thalamic subregions in patients with interictal EM compared with HCs. We found that five thalamic subregions (L-rTtha, L-PPtha, bilateral-Otha, and L-cTtha) exhibited significantly decreased rsFC with the medial stream of pain-related brain regions and DMN, which may modulate emotion and cognitive dysfunction in migraine. More importantly, correlation analysis further validated that the rsFC impairment of thalamic subregions had a close relationship with anxiety and personality traits. Second, for all thalamic subregions and the whole thalamus, there were no significant differences in GMV between patients with EM and HCs (*P* > 0.05, Bonferroni corrected). Finally, there was no intergroup difference in DTI parameters for any thalamic subregion (*P* > 0.05). As a whole, these findings suggest a selective functional dysconnectivity of the thalamic subregions in interictal EM, supporting the idea that the thalamus plays an important role in the thalamocortical pathway in patients with migraine.

The most important finding of this study was the identification of eight distinct patterns of FC, which is consistent with previous functional mappings. Specifically, the rostral temporal thalamus (rTtha) and caudal temporal thalamus (cTtha) are functionally connected to the precuneus gyrus. In addition, functional connections between the occipital thalamus (Otha) and posterior cingulate cortex (PCC) and between Otha and the medial prefrontal cortex (mPFC) were observed in the current study. The mPFC, precuneus and PCC are core regions of the default mode network (DMN), which is particularly responsible for cognitive states in self-referential processing [34]. Additionally, the DMN plays an important role in maintaining pain inhibition efficiency in healthy conditions and in the presence of pain [35]. In line with our findings, Tu et al. found that abnormal posterior thalamus (pulvinar nucleus) dynamic functional network connectivity (dFNC) with the precuneus was significantly correlated with headache frequency in migraine [36]. A recent neuroimaging study showed that migraine patients without aura exhibited significantly reduced FC between the ventral posterior nucleus (VPN) and the left precuneus, right inferior parietal lobule, and right middle frontal gyrus [17].

Moreover, we also found that the posterior parietal thalamus (PPtha) and caudal temporal thalamus (cTtha) were functionally connected to the anterior cingulate cortex (ACC). The posterior nucleus of the thalamus (pTHA) receives projections from the brainstem and relays to the primary and secondary somatosensory cortices (S1 and S2), insula, primary and secondary visual cortices (V1/V2), primary auditory cortex (A1), and ACC [37]. The ACC belongs to the limbic system, connects to the amygdaloid body and is associated with emotional aspects of pain sense, such as evocation, choice of response, foresight, and avoidance of pain stimuli [38, 39]. Traditional investigations have divided ascending nociceptive information into parallel pathways, in which the ACC is part of the medial stream and is involved in processing affective and cognitive aspects of pain [38, 39]. Similarly, previous human and rat studies also provided evidence of altered functional connectivity between the ACC and posterior thalamus, which may contribute to the cutaneous allodynia seen in migraine [40].

It is also interesting to note that the occipital thalamus (Otha) and caudal temporal thalamus (cTtha) were functionally connected to the dorsal lateral prefrontal cortex (DLPFC). The DLPFC has been shown to be involved in the cognitive–affective aspects of processing painful stimuli and has been proposed to exert active control on pain perception by top-down modulation [41]. Previous evidence has suggested the presence of FC changes between the ventroposterolateral (VPL) nucleus, the sensory nucleus of the thalamus, and cortical areas involved in sensory information processing and between the medial dorsal (MD) nucleus, the affective nucleus, and cortical areas involved in affective information processing [42]. Additionally, DLPFC stimulation implied the role of DLPFC in pain modulation, particularly pain tolerance [42].

The correlation analysis in our study revealed that decreased functional connectivity between thalamic subregions and cortical regions was closely related to HAMA and BFI scale scores (extraversion and conscientiousness). However, we did not observe these significant correlations in the HC group. This suggests the important role of the thalamus in emotion and personality traits of pain. Here, we showed, for the first time, that altered FC was densely connected to personality traits in migraine. That is, lower FC changes in migraine patients represented more anxiousness, less extroversion and conscientiousness. However, no rsFCs of any thalamic subregions were correlated with pain-related symptoms. Taken together, these findings suggest that the human thalamus consists of multiple dissociable subregions belonging to different functional networks, mainly located in the medial system of the pain processing pathway and DMN, which serve functions related to emotion and personality trait aspects of pain in EM. The reliable parcellation scheme may provide us with an approach to investigate the thalamus at a subtler level.

Another important finding of this study was that patients with EM presented with decreased GMV in multiple subregions, even when TIV was excluded (*P* < 0.05). However, these significant comparisons were eliminated after Bonferroni correction (*P* > 0.05/16 = 0.003). A multicentre study including 131 patients with migraine and 115 matched HCs used high-resolution T1-weighted MRI scans and discovered that the volumes of several thalamic nuclei were reduced, including the central nuclear complex, anterior nucleus, and lateral dorsal nucleus, which participate in abnormal processing of the affective and cognitive components of pain [19]. Regional analysis also revealed microstructural changes in the ventral posterolateral and ventral posteromedial (VPM) thalamic nuclei compared with HC (*P* < 0.05) [43]. The possible explanation for the discovery of microstructural alterations in migraine may be the larger sample size and excessively liberal statistical modelling. However, no significant correlation between the GMV of the thalamic subregions and clinical scales was detected in this study.

Furthermore, the DTI parameters (FA, RD, MD, and AD) of the thalamic subregions were not significantly different between migraine patients and HCs. Nonetheless, despite these normal results, we found that the majority of DTI parameters in the interictal period of EM patients were lower than those of HCs. These results are in contrast with the abnormal white matter integrity of the bilateral thalami observed in EM between attacks [44].

Several considerations and limitations should be noted when conducting future research. First, the relatively small sample size and lack of some clinical assessments may limit the statistical power in detecting subtle thalamic structural alterations and uncovering potential brain-pain-behaviour relationships. Therefore, a larger sample of patients with migraine and more complete clinical data should be included in future studies to validate our findings. Second, because subjects in the current study compromised migraine with and without aura between attacks, we cannot determine whether our findings can be generalized to all types of migraine. In the future, it will be important to confirm these results in a certain type of subject. Third, although we did not find changes in DTI parameters in the thalamic subregions, differences in patient characteristics (e.g., illness duration, sex, age, education) could potentially affect the structure of the thalamus. Fourth, no correlations were identified between the FCs and any of the pain-related symptoms. We cannot rule out the possibility that there is not a simple linear correlation between altered rsFC and pain-related symptoms. Finally, our cross-sectional design does not allow inference on causality. Longitudinal studies with interventions targeted towards improving clinical symptoms in migraine patients are needed to establish the direction of causality.

## Conclusions

In conclusion, we used rsfMRI and structural techniques to investigate functional connectivity and structural alterations in the thalamus at the subregional level. Our data indicated that patients with EM have impaired functional connectivity but a relatively preserved microstructure in their thalamic subregions relative to HCs. Specifically, functional hypoconnnectivity between the anterior-medial-posterior subregions of the thalamus was discovered in patients with EM, which may contribute to emotion and personality traits of pain in EM. However, the grey matter volume and DTI parameters did not differ between patients with EM and HCs. These findings suggest that selective functional disconnectivity in thalamic subregions may highlight a more sophisticated understanding of the neuropathological mechanism underlying episodic migraine. They also suggest that the thalamus may be a potential pharmacological target for preventive treatment options in migraine.

## Data Availability

The datasets used or analysed during the current study are available from the corresponding authors on reasonable request.

## Abbreviations

fMRI: Resting-state functional magnetic resonance imaging
rsFC: Functional connectivity
GMV: Grey matter volume
DTI: Diffusion tensor imaging
FA: Fractional anisotropy
MD: Mean diffusivity
AD: Axial diffusivity
RD: Radial diffusivity
FD: Framewise displacement
TIV: Total intracranial volume
VAS: Visual analogue scale
HIT-6: Headache Impact Test
HAMA: Hamilton Rating Scale for Anxiety
BDI-II: Beck Depression Inventory-II
MoCA: Montreal Cognitive Assessment
BFI: Big Five Inventory
mPFtha: Medial prefrontal thalamus
mPMtha: Premotor thalamus
Stha: Sensory thalamus
rTtha: Rostral temporal thalamus
PPtha: Posterior parietal thalamus
Otha: Occipital thalamus
cTtha: Caudal temporal thalamus
lPFtha: Lateral prefrontal thalamus

## References

[1] Ashina M, Katsarava Z, Do TP, Buse DC, Pozo-Rosich P, Özge A (2021). Migraine: epidemiology and systems of care. Lancet.

[2] Ashina M, Terwindt GM, Al-Karagholi MA, de Boer I, Lee MJ, Hay DL (2021). Migraine: disease characterisation, biomarkers, and precision medicine. Lancet.

[3] Younis S, Hougaard A, Noseda R, Ashina M (2019). Current understanding of thalamic structure and function in migraine. Cephalalgia.

[4] Ferrari MD, Goadsby PJ, Burstein R, Kurth T, Ayata C, Charles A (2022). Migraine. Nat Rev Dis Primers.

[5] Noseda R, Jakubowski M, Kainz V, Borsook D, Burstein R (2011). Cortical projections of functionally identified thalamic trigeminovascular neurons: implications for migraine headache and its associated symptoms. J Neurosci.

[6] Herrero MT, Barcia C, Navarro JM (2002). Functional anatomy of thalamus and basal ganglia. Childs Nerv Syst.

[7] Fox MD, Raichle ME (2007). Spontaneous fluctuations in brain activity observed with functional magnetic resonance imaging. Nat Rev Neurosci.

[8] Yang Y, Wei K, Zhang H, Hu H, Yan L, Gui W (2022). Identifying functional brain abnormalities in migraine and depression comorbidity. Quant Imaging Med Surg.

[9] Deco G, Kringelbach ML (2014). Great expectations: using whole-brain computational connectomics for understanding neuropsychiatric disorders. Neuron.

[10] Zhu DM, Zhang C, Yang Y, Zhang Y, Zhao W, Zhang B (2020). The relationship between sleep efficiency and clinical symptoms is mediated by brain function in major depressive disorder. J Affect Disord.

[11] Yang Y, Zhu DM, Zhang C, Zhang Y, Wang C, Zhang B et al (2020) Brain Structural and Functional Alterations Specific to Low Sleep Efficiency in Major Depressive Disorder. Front Neurosci. 14(50). 10.3389/fnins.2020.00050. 10.3389/fnins.2020.00050 PMC7005201 32082117

[12] Messina R, Gollion C, Christensen RH, Amin FM (2022). Functional MRI in migraine. Curr Opin Neurol.

[13] Wei HL, Zhou X, Chen YC, Yu YS, Guo X, Zhou GP (2019). Impaired intrinsic functional connectivity between the thalamus and visual cortex in migraine without aura. J Headache Pain.

[14] Amin FM, Hougaard A, Magon S, Sprenger T, Wolfram F, Rostrup E (2018). Altered thalamic connectivity during spontaneous attacks of migraine without aura: A resting-state fMRI study. Cephalalgia.

[15] Wang T, Zhan W, Chen Q, Chen N, Zhang J, Liu Q (2016). Altered resting-state ascending/descending pathways associated with the posterior thalamus in migraine without aura. NeuroReport.

[16] Wang T, Chen N, Zhan W, Liu J, Zhang J, Liu Q (2015). Altered effective connectivity of posterior thalamus in migraine with cutaneous allodynia: a resting-state fMRI study with Granger causality analysis. J Headache Pain.

[17] Qin ZX, Su JJ, He XW, Zhu Q, Cui YY, Zhang JL (2020). Altered resting-state functional connectivity between subregions in the thalamus and cortex in migraine without aura. Eur J Neurol.

[18] Burstein R, Yamamura H, Malick A, Strassman AM (1998). Chemical stimulation of the intracranial dura induces enhanced responses to facial stimulation in brain stem trigeminal neurons. J Neurophysiol.

[19] Magon S, May A, Stankewitz A, Goadsby PJ, Tso AR, Ashina M (2015). Morphological Abnormalities of Thalamic Subnuclei in Migraine: A MulticenterMRI Study at 3 Tesla. J Neurosci.

[20] Taman SE, Kamr WH, Belal TM, Tawfik AI (2021). Diffusion tensor magnetic resonance imaging: is it valuable in the detection of brain microstructural changes in patients having migraine without aura?. Pol J Radiol.

[21] Coppola G, Tinelli E, Lepre C, Iacovelli E, Di Lorenzo C, Di Lorenzo G (2014). Dynamic changes in thalamic microstructure of migraine without aura patients: a diffusion tensor magnetic resonance imaging study. Eur J Neurol.

[22] (2018) Headache Classification Committee of the International Headache Society (IHS) The International Classification of Headache Disorders, 3rd edition. Cephalalgia. 38(1):1–211. 10.1177/0333102417738202. 10.1177/0333102417738202 29368949

[23] Yang M, Rendas-Baum R, Varon SF, Kosinski M (2011). Validation of the Headache Impact Test (HIT-6™) across episodic and chronic migraine. Cephalalgia.

[24] Gallagher EJ, Liebman M, Bijur PE (2001). Prospective validation of clinically important changes in pain severity measured on a visual analog scale. Ann Emerg Med.

[25] Thompson E (2015). Hamilton Rating Scale for Anxiety (HAM-A). Occup Med.

[26] Wang YP, Gorenstein C (2013). Psychometric properties of the Beck Depression Inventory-II: a comprehensive review. Braz J Psychiatry.

[27] Nasreddine ZS, Phillips NA, Bédirian V, Charbonneau S, Whitehead V, Collin I (2005). The Montreal Cognitive Assessment, MoCA: a brief screening tool for mild cognitive impairment. J Am Geriatr Soc.

[28] Soto CJ, John OP (2017). The next Big Five Inventory (BFI-2): Developing and assessing a hierarchical model with 15 facets to enhance bandwidth, fidelity, and predictive power. J Pers Soc Psychol.

[29] Fan L, Li H, Zhuo J, Zhang Y, Wang J, Chen L (2016). The human brainnetome atlas: a new brain atlas based on connectional architecture. Cereb Cortex.

[30] Yan CG, Wang XD, Zuo XN, Zang YF (2016). DPABI: Data Processing & Analysis for (Resting-State) Brain Imaging. Neuroinformatics.

[31] Woolrich MW, Jbabdi S, Patenaude B, Chappell M, Makni S, Behrens T (2009). Bayesian analysis of neuroimaging data in FSL. Neuroimage.

[32] Smith SM, Jenkinson M, Woolrich MW, Beckmann CF, Behrens TE, Johansen-Berg H (2004). Advances in functional and structural MR image analysis and implementation as FSL. Neuroimage.

[33] Bastiani M, Cottaar M, Fitzgibbon SP, Suri S, Alfaro-Almagro F, Sotiropoulos SN (2019). Automated quality control for within and between diffusion MRI studies using a non-parametric framework for movement and distortion correction. Neuroimage.

[34] Buckner RL, Andrews-Hanna JR, and Schacter DL (2008) The brain's default network: anatomy, function, and relevance to disease. Ann N Y Acad Sci 1124:1–38. 10.1196/annals.1440.011. 10.1196/annals.1440.011 18400922

[35] Argaman Y, Kisler LB, Granovsky Y, Coghill RC, Sprecher E, Manor D (2020). The endogenous analgesia signature in the resting brain of healthy adults and migraineurs. J Pain.

[36] Tu Y, Fu Z, Zeng F, Maleki N, Lan L, Li Z (2019). Abnormal thalamocortical network dynamics in migraine. Neurology.

[37] Brennan KC, Pietrobon D (2018). A systems neuroscience approach to migraine. Neuron.

[38] Petrovic P, Ingvar M (2002). Imaging cognitive modulation of pain processing. Pain.

[39] Peyron R, Laurent B, García-Larrea L (2000). Functional imaging of brain responses to pain. A review and meta-analysis. Neurophysiol Clin.

[40] Jia Z, Chen X, Tang W, Zhao D, Yu S (2019). Atypical functional connectivity between the anterior cingulate cortex and other brain regions in a rat model of recurrent headache. Mol Pain.

[41] Lorenz J, Minoshima S, Casey KL (2003). Keeping pain out of mind: the role of the dorsolateral prefrontal cortex in pain modulation. Brain.

[42] Sankarasubramanian V, Cunningham DA, Potter-Baker KA, Beall EB, Roelle SM, Varnerin NM (2017). Transcranial Direct Current Stimulation Targeting Primary Motor Versus Dorsolateral Prefrontal Cortices: Proof-of-Concept Study Investigating Functional Connectivity of Thalamocortical Networks Specific to Sensory-Affective Information Processing. Brain Connect.

[43] Granziera C, Daducci A, Romascano D, Roche A, Helms G, Krueger G (2014). Structural abnormalities in the thalamus of migraineurs with aura: a multiparametric study at 3 T. Hum Brain Mapp.

[44] Coppola G, Di Renzo A, Tinelli E, Lepre C, Di Lorenzo C, Di Lorenzo G (2016). Thalamo-cortical network activity between migraine attacks: Insights from MRI-based microstructural and functional resting-state network correlation analysis. J Headache Pain.

